# Physician decision-making and recommendations for stroke and myocardial infarction treatments in older adults with mild cognitive impairment

**DOI:** 10.1371/journal.pone.0230446

**Published:** 2020-03-17

**Authors:** Deborah A. Levine, Kenneth M. Langa, Angela Fagerlin, Lewis B. Morgenstern, Brahmajee K. Nallamothu, Jane Forman, Andrzej Galecki, Mohammed U. Kabeto, Colleen D. Kollman, Tolu Olorode, Bruno Giordani, Lynda D. Lisabeth, Darin B. Zahuranec

**Affiliations:** 1 Department of Internal Medicine and Cognitive Health Services Research Program, University of Michigan (U-M), Ann Arbor, MI, United States of America; 2 Department of Neurology and Stroke Program, U-M, Ann Arbor, MI, United States of America; 3 Institute for Healthcare Policy and Innovation, U-M, Ann Arbor, MI, United States of America; 4 VA Ann Arbor Healthcare System, Ann Arbor, MI, United States of America; 5 Department of Population Health Sciences, UT and Salt Lake City VA Informatics Decision-Enhancement and Analytic Sciences (IDEAS 2.0) Center for Innovation, University of Utah, Salt Lake City, UT, United States of America; 6 Department of Biostatistics, U-M, Ann Arbor, MI, United States of America; 7 Kollman Research Services, Ann Arbor, MI, United States of America; 8 Department of Psychiatry & Michigan Alzheimer's Disease Center, U-M, Ann Arbor, MI, United States of America; 9 Department of Epidemiology, U-M, Ann Arbor, MI, United States of America; University of Malaya, MALAYSIA

## Abstract

Evidence suggests that older adults with mild cognitive impairment (MCI) might not receive evidence-based treatments. We explored the impact of patient MCI on physician decision-making and recommendations for acute ischemic stroke (AIS) and acute myocardial infarction (AMI) in a pilot concurrent mixed-methods study of physicians recruited from one academic center. The mailed survey included a clinical vignette of AIS or AMI where the patient cognitive status was randomized (normal cognition, MCI, or early-stage dementia). The primary outcome was a composite summary measure of the proportion of guideline-concordant treatments recommended. Linear regression compared the primary outcome across patient cognition groups adjusting for physician characteristics. Semi-structured interviews done with 18 physicians (4 cardiologists, 9 neurologists, 5 internists) using a standard guide. Survey response rate was 72% (82/114) (49/61 neurologists; 33/53 cardiologists). As patient cognition worsened, neurologists recommended less guideline-concordant treatments after AIS (P_trend_<0.001 across patient cognition groups). Cardiologists did not after AMI (P_trend_ = 0.11) in adjusted analyses. Neurologists’ recommendation of guideline-concordant treatments after AIS was non-significantly lower in patients with MCI (composite measure, 0.13 points lower; P = 0.14) and significantly lower in patients with early-stage dementia (0.33 points lower; P<0.001) compared to cognitively normal patients. Interviews identified themes that may explain these findings including physicians assumed patients with MCI, compared with cognitively normal patients, have limited life expectancy, frailty and poor functioning, prefer less treatment, might adhere less to treatment, and have greater risks or burdens from treatment. These results suggest that patient MCI influences physician decision-making and recommendations for AIS and AMI treatments.

## Introduction

Up to 20% of adults ≥65, ~5.4 million Americans, have mild cognitive impairment (MCI), and this number will triple by 2050.[1] MCI is characterized by measurable cognitive impairment that does not severely affect daily functioning.[1] While older patients with MCI, compared to those with normal cognition, are at increased risk for dementia, MCI does not inevitably lead to dementia, even after a decade.[2–4] Many older adults with MCI live ~10 years[4] with good quality of life[5], and face competing health risks of aging, especially cardiovascular disease (CVD).[4] CVD is the leading cause of death and serious morbidity in community-dwelling older adults with and without MCI.[4]

Acute ischemic stroke (AIS) and acute myocardial infarction (AMI) are the most common CVD events.[6] Effective treatments after AIS and AMI reduce death, disability, and increase quality of life.[7–10] Yet evidence suggests older adults with MCI get fewer established, effective treatments after AMI than those with normal cognition.[11, 12] It is unknown how a patient’s MCI influences physician decision-making and recommendations for AIS and AMI treatments.

## Materials and methods

We conducted a pilot concurrent mixed-methods study at one large academic medical center using surveys and semi-structured interviews to explore the influence of MCI on physician decision-making and recommendations for effective treatments for AIS and AMI. We used the information gathered from qualitative interviews to supplement the information that we collected from the surveys.

### Physician survey

We conducted a mailed paper survey of 114 physicians consisting of one clinical vignette of AIS (61 neurologists) or AMI (53 cardiologists). We mailed surveys to all neurologists and cardiologists at a single academic medical center. These specialties were chosen because they make acute reperfusion and revascularization decisions that were queried in the survey. The survey questionnaire used a clinical vignette developed by an interventional cardiologist (BN) and two stroke neurologists (LBM, DBZ) as well as an expert in secondary CVD prevention (DAL). An expert in survey methodology and decision-making (AF) supervised the survey design. Our team has used similarly-designed surveys with clinical vignettes to assess physician decision-making and recommendations in stroke.[13]

The vignette described a 75-year-old patient who had one of three cognitive states: normal cognition, MCI (cognitive difficulties that do not impact daily activities), and early-stage dementia (cognitive difficulties that moderately impact daily activities). We randomized participants to receive one of the three cognitive states which explicitly provided the following data: patient’s cognitive diagnosis (normal cognition, MCI, or early-stage dementia) at a recent clinic visit; Mini-Mental Status Examination score (30/30, 26/30, or 23/30 respectively); and family report on the presence or absence of memory problems and functional limitations (neither, memory problems without functional limitations, or memory problems with functional limitations respectively). The case description of the clinical vignette patient stated that the patient had Medicare insurance, prescription drug coverage, and Blue Cross–Blue Shield supplemental insurance. Neurologists were randomized to one of two clinical vignettes (AIS with high-grade carotid stenosis or AIS with atrial fibrillation at stroke clinic follow-up) using a 3X2 factorial design with two independent variables, one (cognitive state) with three levels and one (CVD event type) with two levels. Cardiologists were randomized to one of three clinical vignettes of AMI (ST-elevation MI; high-risk non-ST-elevation MI, or intermediate-risk non-ST-elevation MI) using a 3X3 factorial design where each of the two independent variables, cognitive state and CVD event type, had three levels. Surveys are available in S1 and S2 Files.

Physicians rated their likelihood of recommending treatments/tests for AIS and AMI for the clinical vignette patient of the survey on a 4-point Likert scale (definitely no, probably no, probably yes, definitely yes). The interventional cardiologist (BN), stroke neurologists (LBM, DBZ), and internist expert in secondary CVD prevention (DAL) selected the most common effective treatments supported by direct randomized controlled trial evidence of substantial benefit of AIS and AMI process measures on clinical outcomes. Effective treatments and tests for AIS were t-PA within three hours[14], carotid artery imaging[10], echocardiogram[10], admission to stroke unit[15], care by inpatient stroke team (because some hospitals lack stroke units)[15], inpatient rehabilitation[16], long-term cardiac monitoring[17], statin[18], and either carotid revascularization for high-grade ipsilateral carotid stenosis[19], or anticoagulation for atrial fibrillation.[20, 21] We did not query specific anti-hypertensive drugs because guidelines do not recommend specific anti-hypertensive drug classes for secondary stroke prevention.[10] Effective treatments for AMI were cardiac catheterization (with revascularization)[22–24], cardiac rehabilitation[25], beta-blocker[26, 27], statin[28], and ACE inhibitor[29–33]. We did not query physician recommendations for anti-platelets because aspirin use is high and varies relatively little by physician practice nor the selection of non-aspirin anti-platelet drugs because their indication might depend on CVD event features and treatment details that we did not provide in the vignette.

Physicians also rated their likelihood of asking the vignette patient’s preferences for receiving each of the treatments. Physicians reported personal/practice characteristics (age, years since medical school graduation, race/ethnicity, gender, board certification, outpatient work, academic setting, close family/friend with dementia, number of patients with AIS or AMI cared for in past 12 months). Physicians attitudes toward the patient (likelihood that patient will miss follow-up appointments, participate in treatment, comply with therapy, and sue for malpractice) were assessed based on questions from the literature.[34] Physicians also estimated the patient’s predicted 5-year risks of dementia, AIS, and AMI. We mailed the survey once with an unconditional incentive of $35 cash included in the envelope because surveys with unconditional incentives (incentive is provided regardless of survey questionnaire completion) have higher response rates than surveys with conditional incentives (incentives provided after survey response).[35, 36]

### Physician interviews

We conducted a descriptive qualitative pilot study using in-person, semi-structured interviews to explore the influence of MCI on physician decision-making and recommendations for CVD treatments. The pilot study population consisted of 18 physicians who practiced in one of three specialties (neurology, cardiology, and internal medicine) at a large academic medical center. We chose these specialties because they care for most AIS and AMI cases. We used purposeful sampling to identify and select information-rich subjects efficiently.[37] The Principal Investigator (DAL) sent an email invitation and recruited participants from a purposeful sample of 39 expert physicians who were selected based on their experience, willingness to participate, and communication skills.[37] By design, we interviewed a minimum of 3–5 physicians in each specialty. Participants received $100 incentive after completing the interview.

A trained qualitative researcher (CDK) used a standard guide consisting of questions regarding physician experience caring for patients with MCI and gradually focused on how patient MCI influences physician decision-making and recommendations for AIS and AMI treatments which was the focus of the pilot study. The key questions of interest from the interviews were: “Has the fact that a patient has mild cognitive impairment influenced how you treated him or her for heart attack/stroke? If yes, how?” Neurologists and half of the internists were asked about stroke while cardiologists and the remaining half of the internists were asked about heart attack. We used specific treatments for heart attack and stroke as probes. The interview guide is available in the S3 File. The interviewer did not know the physician participants.

We discontinued data collection after 18 physicians were interviewed because thematic saturation was achieved based on the inductive approach; specifically, interviews did not identify new codes for how patient MCI influenced physician decision-making or recommendations for AIS or AMI. [38] A professional transcriptionist transcribed the interviews. De-identified transcripts were uploaded into Dedoose, a qualitative and mixed methods software package, for analysis (http://www.dedoose.com/)[39].

### Survey analysis

The primary outcome was a composite summary measure, calculated as the average of physicians’ individual recommendations for all guideline-concordant treatments (score range 1–4). We first tested for difference in the composite summary measures across the three cognitive status groups using Wilcoxon rank-sum non-parametric test. We then tested for differences in the outcome between patient cognition groups stratified by physician specialty using linear regression before and after adjusting for physician characteristics. We also tested for a trend in the outcome across the three patient cognitive groups using an extension of Wilcoxon rank-sum non-parametric test[40] for unadjusted and orthogonal polynomial contrasts test for adjusted model. Physician age, gender, and having close family/friend with dementia as well as CVD event type (AIS type: stroke with atrial fibrillation vs. stroke with high-grade carotid stenosis; AMI type: STEMI vs. high-risk NSTEMI vs intermediate-risk NSTEMI) were included in all models regardless of statistical significance. Other variables that did not reach statistical significance (defined as P<0.05) were not included in models; these were years since medical school graduation, board certification, and number of patients with AIS or AMI cared for in past 12 months.

We tested for differences in physicians’ recommendations for individual treatments using Fisher’s exact test. We compared “definitely yes” versus remaining responses for physician recommendations for individual treatments because, for most treatments recommended by cardiologists, the two categories “definitely no”, “probably no” have very low frequencies of the order of 0–3 and for this reason they were combined with the next adjacent category “probably yes”. We compared physicians’ responses to other questions using Fisher’s exact test. We tested for differences in physicians' predicted 5-year risks of AIS, AMI, and dementia across the clinical vignette patient’s cognitive status by physician specialty using unadjusted linear regression.

### Interview analysis

For the qualitative component of the evaluation, we used a descriptive qualitative methodology grounded in a naturalist philosophy, wherein the goal is to be “data-near,” reporting findings in their everyday terms, rather than more highly theorized.[41] The underlying epistemology is subjectivism.[42] In using inductive qualitative content analysis[43] as our approach to coding, we accepted data as representing our participants' subjective perceptions, and our role as researchers in using our skills to interpret the phenomenon at hand based on these perceptions. This approach, as well as the pragmatic paradigm underlying our mixed methods approach, supports our goal of producing concrete findings for real-world practice.[44]

We performed qualitative content analysis to identify unifying and recurrent themes of the interviews using the Dedoose web application (http://www.dedoose.com/). The coding team consisted of a vascular neurologist (DBZ), internal medicine physician (DAL), qualitative researchers, and study staff. First, the coding team read through the first several transcripts to identify factors that might explain why physicians might treat patients with MCI differently than cognitively normal patients. The team then created and defined codes corresponding to these factors. Codes were not pre-specified, but emerged from the data and iteratively discussed by the coding team. Codes were organized into seven main themes. Transcripts were coded with themes (not original factor codes) in an iterative process that included discussing and expanding themes. Findings were developed through review of the code reports. Inter-coder reliability was established through double-coding one third of the interviews and discussing discrepancies. The University of Michigan Institutional Review Board approved the study.

### Data sharing

The analytic dataset (“the minimum data set”) of the survey data is available (https://github.com/deblevine/MADCpilotsurveydataset). We cannot make the interview transcripts available because we did not obtain informed consent to publicly share the transcript data from participants.

## Results

### Survey results

The survey response rate was 72% (82/114) (49/61 neurologists; 33/53 cardiologists). **Table 1** presents physician characteristics.

**Table 1:** Physician characteristics.

Characteristics	N (%)
**Survey Physicians (n = 82)**	
Age, median (interquartile range)	44 (38–54)
Years since medical school graduation, median (interquartile range)	18 (12–28)
White race	65 (80)
Hispanic ethnicity	1 (1)
Female gender	19 (23)
Practice specialty	
Cardiology	33 (40)
Neurology	49 (60)
Board certification	82 (100)
Close family/friend with dementia	50 (62)
Number of patients with acute ischemic stroke or myocardial infarction cared for in past 12 months	
None	5 (6)
1–10	17 (21)
11–20	17 (21)
>20	42 (52)
**Interview Physicians (n = 18)**	
Years since medical school graduation, (interquartile range)	16 (11 to 25)
Race	
Caucasian	14 (78)
Asian	4 (22)
Female gender	6 (33)
Specialty	
Cardiology	4 (22)
Internal Medicine	5 (28)
Neurology	9 (50)
Board certification	18 (100)

#### Unadjusted physicians' recommendations of guideline-concordant treatments for AIS and AMI by cognitive status of clinical vignette patient

As patient cognition worsened, neurologists were less likely to recommend guideline-concordant treatments after AIS (P<0.001 for trend for composite summary measure across patient cognitive groups) (**Table 2**). Neurologists recommended less carotid imaging (P_trend_ = 0.001), echocardiogram (P_trend_ = 0.02), carotid revascularization or anticoagulation(P_trend_ = 0.003), and inpatient rehabilitation (P_trend_ = 0.005) after AIS as patient cognition worsened (**Table 2**). Cardiologists’ recommendations for guideline-concordant treatments after AMI did not change as patient cognition worsened (P = 0.27 for trend for composite summary measure). Cardiologists recommended a similar average number of guideline-concordant treatments in cognitively normal patients (mean composite summary score, 3.9 treatments [standard deviation, 0.13]), those with MCI (3.9 treatments [0.10]), and those with early-stage dementia (3.9 treatments [0.33]).

**Table 2:** Physicians' recommendations of guideline-concordant treatments for acute ischemic stroke and acute myocardial infarction by cognitive status of clinical vignette patient.

	Cognitive Status of Clinical Vignette Patient			
Treatment/Test, n (%)	Normal Cognition	MCI	Early-stage Dementia	P value*
**Acute ischemic stroke (neurologists, n = 49)**				
	**(n = 19)**	**(n = 15)**	**(n = 15)**	
Composite summary score, mean (SD)	3.9 (0.14)	3.7 (0.22)	3.5 (0.31)	<0.001**
t-PA within 3 hours	17 (89)	12 (80)	12 (80)	0.70
Carotid artery imaging	19 (100)	14 (93)	8 (53)	0.001
Echocardiogram	18 (95)	14 (93)	9 (60)	0.02
Admission to stroke unit	19 (100)	15 (100)	14 (93)	0.61
Care by an inpatient stroke team	19 (100)	15 (100)	14 (93)	0.61
Carotid revascularization or anticoagulation (depending on stroke type/etiology)	17 (89)	10 (67)	5 (33)	0.003
Inpatient rehabilitation	18 (95)	12 (80)	7 (47)	0.005
Long-term cardiac monitoring	12 (63)	5 (33)	7 (47)	0.25
Statin	16 (84)	12 (80)	10 (67)	0.58
**Acute myocardial infarction (cardiologists, n = 33)**				
	**(n = 13)**	**(n = 8)**	**(n = 12)**	
Composite summary score, mean (SD)	3.9 (0.13)	3.9 (0.10)	3.7 (0.33)	0.27**
Cardiac catheterization	9 (69)	8 (100)	6 (50)	0.05
Cardiac rehabilitation	12 (92)	5 (63)	8 (67)	0.23
Beta-blocker	11 (85)	8 (100)	10 (83)	0.65
Statin	13 (100)	8 (100)	11 (92)	0.61
ACE inhibitor	13 (100)	8 (100)	10 (83)	0.18

The composite summary measure was calculated as the average of physicians’ individual recommendations for all effective treatments (score range 1–4). For individual treatments, responses categorized as definitely yes versus others (definitely no, probably no, probably yes).

*P-value from Fisher’s exact test.

**P-value for trend across 3 cognitive status groups using an extension of Wilcoxon rank-sum non-parametric test.

#### Adjusted physicians' recommendations of guideline-concordant treatments for AIS and AMI by cognitive status of clinical vignette patient

After adjusting for physician factors and stroke type, neurologists remained less likely to recommend guideline-concordant treatments/tests after AIS as patient cognition worsened (P_trend<_0.001) (**Table 3**). Neurologists’ recommendation of guideline-concordant treatments/tests after AIS was non-significantly lower in patients with pre-existing MCI (composite summary measure, 0.13 points lower; P = 0.10) and significantly lower in patients with pre-existing early-stage dementia (0.35 points lower; P<0.001) compared to cognitively normal patients (**Table 3**). When the outcome was restricted to the 4 treatments only (IV t-PA within 3 hours, inpatient rehabilitation, statin, and either carotid revascularization for high-grade ipsilateral carotid stenosis or anticoagulation for atrial fibrillation) without the tests, results were similar (**Table 3**). Neurologists recommended less guideline-concordant care to patients with early-stage dementia than to patients with MCI when the outcome was the combination of nine treatments and tests (adjusted difference, -0.22 points [95%CI, -0.39, -0.05]; P = 0.01) and the outcome was limited to the four treatments only (adjusted difference, -0.25 points [95%CI, -0.47, -0.03]; P = 0.03). Neurologists who had a family/friend with dementia recommended more guideline-concordant treatments when the outcome was the combination of nine treatments and tests (adjusted difference in the composite summary measure, 0.14 points lower [95%CI, -0.002, 0.28]; P = 0.05) and results were similar when the outcome was limited to the 4 treatments only (**Table 3**). No other physician factors were significantly associated with recommendations for AIS treatment.

**Table 3:** Differences (95% Confidence Intervals) in composite summary measure of physicians' recommendations of guideline-concordant treatments and tests for acute ischemic stroke and acute myocardial infarction by cognitive status of clinical vignette patient.

Cognitive Status of Clinical Vignette Patient	Acute ischemic stroke (neurologists, n = 49) based on 9 treatments and tests		Acute ischemic stroke (neurologists, n = 49) based on 4 treatments		Acute myocardial infarction (cardiologists, n = 33)	
	Unadjusted	Adjusted	Unadjusted	Adjusted	Unadjusted	Adjusted
**Patient MCI vs normal cognition**	-0.16 (-0.31, 0.003) P = 0.06	-0.13 (-0.29, 0.03) P = 0.10	0.16 (-0.36, 0.03) P = 0.10	-0.14 (-0.35, 0.07) P = 0.19	0.03 (-0.17, 0.24) P = 0.75	0.01 (-0.27, 0.29) P = 0.96
**Patient early-stage dementia vs normal cognition**	-0.34 (-0.50, -0.18) P<0.001	-0.35 (-0.50, -0.20) P<0.001	-0.39 (-0.59, -0.20) P<0.001	-0.39 (-0.58, -0.19) P<0.001	-0.16 (-0.34, 0.02) P = 0.09	-0.21 (-0.46, 0.05) P = 0.11
**Age per 1-year increase**		-0.001 (-0.008, 0.006) P = 0.82		-0.006 (-0.01, 0.003) P = 0.20		-0.003 (-0.01, 0.008) P = 0.60
**Women vs men**		0.09 (-0.05, 0.23) P = 0.19		0.12 (-0.06, 0.31) P = 0.19		-0.04 (-0.33, 0.25) P = 0.76
**Whites vs non-Whites**		-0.09 (-0.26, 0.07) P = 0.26		-0.01 (-0.23, 0.20) P = 0.90		0.13 (-0.13, 0.39) P = 0.32
**Having close family/friend with dementia**		0.14 (-0.002, 0.28) P = 0.05		0.18 (-0.004, 0.37) P = 0.06		-0.09 (-0.31, 0.12) P = 0.39
**AIS type**						N/A
**Atrial fibrillation versus carotid stenosis**		0.10 (-0.02, 0.23) P = 0.11		0.09 (-0.08, 0.25) P = 0.29		
**AMI type**		N/A		N/A		
**High-risk NSTEMI vs STEMI**						0.11 (-0.11, 0.33) P = 0.32
**Intermediate risk NSTEMI vs STEMI**						0.05 (-0.21, 0.31) P = 0.68
P value for trend across 3 cognitive groups*	<0.001	<0.001	<0.001	<0.001	0.09	0.11

Abbreviations: AIS is acute ischemic stroke. AMI is acute myocardial infarction. NSTEMI is non-ST-elevation myocardial infarction. STEMI is ST-elevation-myocardial infarction. N/A is not applicable.

Linear regression models adjusted for physician age, gender, and having close family/friend with dementia as well as stroke/AMI type (stroke type: stroke with atrial fibrillation vs. stroke with high-grade carotid stenosis; AMI type: STEMI vs. high-risk NSTEMI vs intermediate-risk NSTEMI).

The nine stroke treatments and tests were IV t-PA within 3 hours, inpatient rehabilitation, statin, either carotid revascularization for high-grade ipsilateral carotid stenosis or anticoagulation for atrial fibrillation, carotid artery imaging, echocardiogram, admission to stroke unit, care by an inpatient stroke team, and long-term cardiac monitoring. The four stroke treatments were IV t-PA within 3 hours, inpatient rehabilitation, statin, and either carotid revascularization for high-grade ipsilateral carotid stenosis or anticoagulation for atrial fibrillation

*P-value for trend across 3 cognitive groups using Orthogonal polynomial contrasts test.

After adjusting for physician factors and AMI type, cardiologists’ recommendations for guideline-concordant treatments after AMI did not differ as patient cognition worsened (P_trend_ = 0.11)(**Table 3**). Cardiologists’ recommendation of guideline-concordant treatments/tests after AIS was similar in patients with pre-existing MCI (adjusted difference in composite summary measure, 0.01 points; P = 0.96) and non-significantly lower in patients with pre-existing early-stage dementia (0.21 points lower; P = 0.11) compared to cognitively normal patients (**Table 3**). Cardiologists recommended fewer guideline-concordant treatments to patients with early-stage dementia than to patients with MCI (adjusted difference, -0.21 points [95%CI, -0.45, 0.02]; P = 0.08). No physician factors were significantly associated with recommendations for AMI treatment.

#### Physicians' predicted risks of dementia, AIS, and AMI by cognitive status of clinical vignette patient

Neurologists predicted that MCI patients had significantly higher risk of dementia compared with cognitively normal patients (**Table 4**). Cardiologists predicted that MCI patients had significantly higher risks of dementia, AIS, and AMI than cognitively normal patients.

**Table 4:** Physicians' predicted 5-year risks of clinical vignette patient by physician specialty.

	Cognitive Status of Clinical Vignette Patient				
Predicted Five-Year Risks	Normal Cognition	MCI	Difference MCI vs Normal Cognition Estimate (SE) P-value*	Early-stage Dementia	Difference Early-stage Dementia vs Normal Cognition Estimate (SE) P-value*
	**Acute ischemic stroke (neurologists, n = 49)**				
	**(n = 19)**	**(n = 15)**		**(n = 15)**	
Probability of dementia	21.1% (15.9)	52.0% (27.8)	30.9% (7.6) P<0.001	NA	NA
Probability of AIS	20.9% (13.9)	28.5% (16.9)	7.6% (5.3) P = 0.16	29.1% (20.2)	8.2% (5.9) P = 0.17
Probability of AMI	20.9% (14.2)	29.0% (19.4)	8.1% (5.8) P = 0.17	22.0% (14.2)	1.1% (4.9) P = 0.82
**Acute myocardial infarction (cardiologists, n = 33)**					
	**(n = 13)**	**(n = 8)**		**(n = 12)**	
Probability of dementia	10.6% (9.2)	51.9% (20.7)	41.3% (6.5) P<0.001	NA	NA
Probability of AIS	7.5% (4.1)	18.1% (13.9)	10.7% (4.1) P = 0.02	9.4% (7.3)	2.0% (2.3) 0.41
Probability of AMI	15.4% (9.5)	27.5% (14.1)	12.1% (5.1) P = 0.03	21.3% (16.5)	5.9% (5.3) 0.28

*Results from two-sample t-test. Normal cognition is referent. SE, standard error.

#### Physicians' attitudes by cognitive status of clinical vignette patient

We also assessed physician attitudes toward the clinical vignette patients by cognitive status. Since neurologists and cardiologists received the same questions, we present the results for the combined group of both physician specialties. Both physician groups rated patients with MCI and those with early-stage dementia as more likely to miss follow-up appointments (P = 0.01), and less likely to participate in treatment (P = 0.03) and comply with treatment (P = 0.06) than cognitively normal patients but not more likely to sue for malpractice (P = 0.30).

#### Physicians' elicitation of treatment preferences by cognitive status of clinical vignette patient

We also assessed whether physicians would elicit the treatment preferences of the clinical vignette patient differently based on the cognitive status of the patient. As patient cognition worsened, neurologists were somewhat more likely to elicit patient preferences for receiving statin (P = 0.09) but not thrombolysis (P = 0.27), gastrostomy tube (P = 0.23), inpatient rehabilitation (P = 0.25), oral anticoagulation for atrial fibrillation (P = 0.36), or carotid revascularization (P = 0.58). As patient cognition worsened, cardiologists were somewhat more likely to elicit patient preferences for receiving statin (P = 0.04), beta-blocker (P = 0.08), and ACE inhibitor (P = 0.10) but not cardiac catheterization (P = 0.69), coronary artery bypass graft surgery (P = 1.00), and cardiac rehabilitation (P = 1.00).

### Interview themes

We interviewed 18 physicians (9 neurologists, 4 cardiologists, and 5 internists). All physicians but one reported that MCI might influence their decision-making and recommendations for AIS or AMI treatment. We identified six themes in the interviews of physicians across the three specialties of cardiology, neurology, and internal medicine. Representative quotations from the physician interviews can be found in **Table 5**.

**Table 5:** Interview themes and exemplar quotes for how patient MCI might influence physician decision-making and recommendations for treatments after stroke and myocardial infarction.

Theme	Exemplar Quotes
Physicians believed that patient MCI influences decision-making and recommendations for acute stroke and acute myocardial infarction treatments with more severe MCI having a greater effect than less severe MCI.	“People with mild cognitive impairment, it kind of depends on where on the spectrum they are and sometimes it’s also how aggressive they want to be. So I mean, it probably factors in, probably not consciously or overtly as much as it would in a patient with dementia.” (physician 7, neurologist)
	“Yes, it really depends on the severity of the MCI…It might influence whether we do that test at all, whether we do a cardiac cath at all…” (physician 16, internist)
	“If there is a clear-cut indication [for oral anticoagulation], it [MCI] shouldn’t matter. Now if you are starting to become closer to dementia then that is another consideration.” (physician 14, neurologist)
	“Let’s say if they’re mild cognitive impairment was quite mild then it might be suitable for them to have aggressive treatment by a cardiologist.” (physician 16, internist)
Physicians assumed that patients with MCI have shortened life expectancy and poor prognosis.	“We know that patients with MCI have a reduced lifespan compared to someone who has no cognitive impairment” (physician 10, cardiologist).
	“I know that many patients with MCI stay in a state of MCI, but there is probably 5% to 10% that progress to dementia per year, so it might make me less likely to do a test that might lead to a more invasive procedure in the future…. do MCI patients generally have the same life expectancy at 70 as someone without cognitive impairment?” (physician 13, neurologist)
Physicians assumed that patients with MCI are frailer and have poorer functional status than cognitively normal patients.	“Their baseline cognitive status in the sense of their baseline functional ability…I think probably the right term is frailty.” (physician 3, internist)
	“So it’s the function piece that kind of sometimes gets to be concerning.” (physician 7, neurologist)
	Regarding the recommendation for intravenous thrombolysis for stroke, “Maybe. So if they have mild cognitive–it’s the same spectrum. Assuming…somebody’s pretty much independent, then no, but if it’s an older, more frail person, then yes.” (physician 3, internist)
Physicians assumed that patients with MCI might not adhere to treatment.	“I worry about patients not complying with the diet or taking too many or not enough of medicines like Warfarin, in particular” (physician 8, internist).
	Regarding the recommendation for cardiac rehabilitation after AMI, “Can they follow instructions?” (physician 16, internist).
	“…the American Heart Association guidelines actually say that if you don’t think a patient is going to be able to comply with dual antiplatelet therapy there’s actually a harm associated with putting a stent in their coronary arteries and so the stakes are fairly high with figuring out is somebody going to be able to take their medications. And I think people with mild cognitive impairment, that’s a big question that’s much more difficult to answer.” (physician 8, internist)
	“I might reconsider whether if somebody had a lot of memory problems if memory was a big component and they were forgetting their medicines, the more complicated medicine with higher risk may not be a good choice so Coumadin.” (physician 14, neurologist)
Physicians made assumptions that MCI is associated with patient/family preferences for less intensive treatment.	“…well informed patients …who have an extremely high priority on their cognitive function and if they’re aware they have cognitive impairment based on a number of things—based on, let’s say, geriatrics, based on the feelings of their spouse about how they’re repeating themselves or certain things about asking the same questions, and they’re aware of cognitive impairment, they’re aware the imaging of their brain by CT or MRI was not perfect, and if they were to think about getting bypass surgery they might be aware that we’re not sure if something about bypass surgery or the sedation that’s required in bypass surgery affects that. That might make them more reluctant to consider that option.” (physician 16, internist)
	“I just have concerns, frankly, that the patient and family would choose against it [surgery] because they don’t understand what it means" (physician 12, internist).
Physicians worried that patients with MCI have greater risks or burdens from treatment.	“Invasive procedures, I think there’s a gray spectrum there and for that reason, I think those are conversations where I would want to take into account a patient’s baseline cognitive status and their family and their living situation before making a decision.” (physician 3, internist)
	“I suppose that I might be more inclined to consult PM&R earlier in somebody’s hospital stay if they had a new acute focal weakness from a stroke and the complete absence of any cognitive deficits, versus if they had MCI, I might be more inclined to wait to hear what PT and OT thought, and if they thought that the person would be a good candidate for rehab, then consult them. …I probably am more inclined to consult PM&R more quickly if somebody has normal cognition and has a new neurological deficit than if they have impaired cognition and a new neurologic deficit.” (physician 4, neurologist)

Abbreviations: PM&R is Physical Medicine and Rehabilitation. PT is physical therapy. OT is occupational therapy.

Physicians believed that patient MCI influences decision-making and recommendations for AIS and AMI treatments with more severe MCI having a greater effect than less severe MCI. Many physicians assumed that patients with MCI have shorter life expectancy, are frailer, and have poorer functional status than cognitively normal patients. Not only did physicians assume that patients with MCI might not adhere to treatment, but they also believed that patients with MCI have greater risks or burdens from treatment than cognitively normal patients. Patient MCI influenced physician decision-making and recommendations for both invasive, burdensome treatments as well as non-invasive treatments. Physicians also made assumptions that patients with MCI and their families prefer less intensive treatment.

In addition to these themes, some physicians discussed the challenges in caring for patients with MCI. Physicians expressed concern about the extent to which patients with MCI understand the benefits and risks of the treatment being considered. One internist (physician 8) said, “I think one of the key things is shared decision-making and the risk benefit discussions …you have someone who has a heart attack and you need to make a recommendation to them about whether or not they go forward with a left heart catheterization, and there is risk associated with that, you know, risk of vascular injury, risk of renal failure from the contrast agent that’s given. And you know, with mild cognitive impairment it can be a challenge to, you know, help sort out how much of that risk do they really understand.” Although we defined MCI at the beginning of the interview, some physicians were still unaware of the definition of MCI and conflated MCI with dementia. One cardiologist (participant 9) asked, “What’s the difference?”.

## Discussion

As patient cognition worsened, neurologists recommended less guideline-concordant treatments after AIS. Patient MCI had a modest effect and patient dementia had a stronger effect on neurologists’ treatment recommendations after AIS. Cardiologists recommended less guideline-concordant treatment after AMI for patients with early-stage dementia but not MCI compared to those with normal cognition.

Compared to the population of older adults with dementia, relatively little is known about physician decision-making and recommendations for guideline-concordant treatment in older adults with MCI. Previous studies have shown that pre-existing MCI was associated with lower likelihood of receiving invasive treatments (e.g., cardiac catheterization and coronary revascularization) after AMI. [11, 12] The association between patient MCI and receipt of non-invasive treatments such as cardiac rehabilitation is unclear but one study has found lower likelihood of physician referral and patient participation in cardiac rehabilitation.[11] Differences in use of secondary preventive medications after AIS and AMI are also unclear with one study finding similar use of AMI medications by cognitive status.[11]

Patients with MCI might get fewer invasive treatments after AIS and AMI because of physician recommendations, patient/care partner preferences, or a combination of both. Our survey results suggest that physicians might be concerned that cognitively impaired patients are more likely to miss follow-up appointments and less likely to participate in or comply with treatment than cognitively normal patients. Our findings that physicians might elicit patient preferences for secondary CVD prevention medication *more* in cognitively impaired patients than cognitively normal patients suggest that, as patient cognition worsens, physicians might engage in *more* shared decision-making for non-invasive treatments especially those perceived to have less benefit (e.g., statins in adults older than 75). Physicians might also recommend invasive treatments less frequently to patients with MCI owing to concerns about treatment risks. Yet, studies suggest that patients with MCI and cognitively normal patients have similar risks and outcomes of CVD treatments including thrombolysis for stroke [45, 46] and coronary revascularization.[47]

The interviews identified additional potential reasons why physicians might recommend fewer guideline-concordant treatments to patients with pre-existing MCI than to cognitively normal patients. Physicians might conflate MCI with dementia or overestimate the risk of dementia, consistent with research showing that people tend to overestimate the likelihood of rare events and underestimate the likelihood of common events.[48] The risk of conversion from MCI to dementia varies by population ranging from 3–15% per year with lower risk in community-based samples and higher risk in samples from Alzheimer’s Disease clinics and research centers.[2, 3, 49] Although patients with MCI have an increased risk of progressing to dementia, it is not inevitable that they will develop dementia, even after a decade.[2–4] Our survey results suggest that neurologists might have recommended fewer guideline-concordant treatments after AIS in patients with MCI because neurologists predicted that the patients with MCI had significantly higher risk of dementia but not CVD.

Physicians also assumed that patients with MCI have poor prognosis and limited life expectancy. Yet, in a study of community-dwelling, older adults with MCI, average life expectancy was nearly 10 years and CVD caused substantially more deaths than dementia did (38% vs. 3%).[4] Physicians assumed that patients with MCI are frailer and have poorer functional status than cognitively normal patients. The association between patient MCI and patient frailty is unclear but some have found that patients who are classified as frail are not more likely to have MCI compared to the overall population.[50] Physicians also might assume that patients with MCI and their families prefer less intensive treatment. These physicians’ assumptions about MCI patients might not be evidence-based. We were unable to find evidence that patients with MCI want less treatment than cognitively normal patients.

Our study has limitations. We studied physicians at one academic medical center. Results may not generalize to non-academic physicians. Each physician respondent saw one patient vignette with one cognitive state. Our pilot survey findings warrant confirmation in a larger study. We did not examine whether treatment factors (e.g., invasiveness, risks) modify the effect of patient MCI on physician recommendations for treatment because the sample size for the surveys was small. The pilot study potentially did not detect differences in physician recommendations for treatments between groups based on patient cognitive status because the study lacked statistical power and precision and also cardiologists tended to definitely or probably recommend most treatments consistent with high-quality care. Currently, we are conducting a larger survey of cardiologists, neurologists, and generalist physicians (physicians in internal medicine, family medicine, and geriatrics) to further assess the influence of patient MCI on physician decision-making based on the results of this pilot study. Observed differences in recommendations might reflect individual differences in clinical practice rather than causal associations between patient MCI and physician recommendations. We did not examine themes by physician specialty because the sample size was small. Although the interview sample was small, thematic saturation was achieved.

Our results suggest that patient MCI might influence physician decision-making and recommendations for AIS and AMI treatments. Further research is needed to better understand how physician recommendations and attitudes as well as patient and care partner preferences might contribute to underuse of effective CVD treatments in patients with MCI. With greater emphasis on shared decision-making between patient and physician, a key concern is that patients with MCI get the care that they would want if properly informed. This is important because the number of older adults diagnosed with MCI likely will increase because the Affordable Care Act mandated coverage of an assessment of cognitive impairment as part of the annual wellness benefit for Medicare beneficiaries.[51] Our findings also suggest that efforts to improve neurologists’ and cardiologists’ understanding of MCI and its prognosis (i.e., dementia is not inevitable) might be warranted.

## Conclusions

The survey results are consistent with patient MCI influencing neurologist decision-making and recommendations for AIS treatments but not those of cardiologists for AMI treatments. The interview results suggest potential reasons why physicians might recommend less treatment to patients with MCI including assumptions that patients with MCI have shortened life expectancy/poor prognosis and frailty/poor functional status, might not adhere to treatment, have greater risks or burdens from treatment, and might prefer less intensive treatment. Research is needed to improve understanding of how physician recommendations may contribute to underuse of effective CVD treatments in patients with MCI.

## Supporting information

S1 File Physician perspectives on myocardial infarction treatment. (PDF)

S2 File Physician perspectives on stroke treatment. (PDF)

S3 File Physician interview guide. (PDF)
