# Supplementary material for: Physician decision-making and recommendations for stroke and myocardial infarction treatments in older adults with mild cognitive impairment
Source: PLoS One. 2020 Mar 17;15(3):e0230446. doi: 10.1371/journal.pone.0230446 (PMC7077853; doi:10.1371/journal.pone.0230446)
Supplement: S1 File — (PDF) [file pone.0230446.s001.pdf]

Physician Perspectives on Myocardial Infarction Treatment

Sponsored by:

Michigan Alzheimer's Disease Center - Pilot Project Program

Return to:

Dr. Deborah Levine
Departments of Medicine & Neurology
2800 Plymouth Road, Building 16-430W
Ann Arbor, Michigan 48109-2800

Questions? Please call (734) 936-5216 or email deblevin@umich.edu

This study is funded by a grant from the Michigan Alzheimer's Disease Center - Pilot Project Program and has been approved by the Institutional Review Board of the University of Michigan (IRB number HUM00096544).

3B

Physician Perspectives on Myocardial Infarction (MI) Treatment

Thank you for taking the time to complete this survey. We are interested in learning **how doctors make recommendations for patients with myocardial infarction (MI) and perceive patients' health risks**. Your answers are **important** to us.

This survey includes **one example case of MI**. We are specifically interested in **your opinion** on the case, and what you would recommend to the patient and family. There is **no right or wrong answers**, so please answer as you would if the patient in the case were one of your own. We realize that you may ordinarily use additional information in your clinical decision making that is not presented in the case. Please provide your **one best answer to each question** based on the information provided.

Your answers will be kept **confidential and anonymous.**

When you are completing the survey, feel free to **mark the survey with any comments** about questions or answers that are difficult. There is also **space at the end** to write comments.

Section A: Case

Please read the brief clinical scenario below and answer the questions that follow.

Case

A 75 year-old man presents after two 60-minute episodes of intense substernal chest pain at rest in the last 24 hours. The chest pain was not pleuritic.

His past medical history is significant for hypertension, current smoking, and osteoarthritis. At a recent clinic visit, he was diagnosed with mild cognitive impairment (Mini-Mental Status Examination score was 26 out of 30). His family reports mild difficulty remembering recent conversations, dates, and appointments. He functions independently and does not require help with dressing, bathing, grocery shopping, or taking medicines, but he uses reminders more.

He takes a daily aspirin. He has a family history of premature coronary artery disease. He lives at home with his wife. He is a retired auto worker with Medicare insurance, prescription drug coverage, and Blue Cross–Blue Shield supplemental insurance.

Examination reveals a normal temperature with a blood pressure of 150/68 and a pulse of 90, normal respiratory rate and oxygen saturation of 100%. His exam is unremarkable with no signs or symptoms of heart failure. Kidney function is normal

ECG shows new 1 mm ST elevations in leads V1 to V3. Cardiac biomarkers are elevated. Troponin I is 8.0 ng/mL (normal is 0.00 to 0.29 ng/mL).

He is diagnosed with a non-ST elevation MI. His TIMI risk score is 5 (age ≥75, hypertension, anterior ST elevation).

---

1. What is the likelihood that the patient will benefit from an early invasive strategy with cardiac catheterization and possible revascularization procedure (coronary angioplasty or coronary-artery bypass surgery)?

Very unlikely	Unlikely	Likely	Very likely
☐	☐	☐	☐

2. In a patient like this, how likely would you be to…

	Definitely no	Probably no	Probably yes	Definitely yes
Recommend cardiac catheterization	☐	☐	☐	☐

Now imagine that the patient underwent cardiac catheterization. A stent was placed in the mid-left anterior descending coronary artery. His echocardiogram shows left ventricular systolic function of 40%. He is being discharged home.

3. In a patient like this, how likely would you be to…

	Definitely no	Probably no	Probably yes	Definitely yes
a. Recommend cardiac rehabilitation	☐	☐	☐	☐
b. Recommend beta-blocker	☐	☐	☐	☐
c. Recommend statin	☐	☐	☐	☐
d. Recommend ACE inhibitor	☐	☐	☐	☐

4. For the following questions, we would like to know more about your assessment of the patient. Given the limited information that you have, we would like you to make predictions about what you think would happen with this patient.

	Very unlikely	Unlikely	Likely	Very likely
a. What is the likelihood that the patient will miss follow-up appointments?	☐	☐	☐	☐
b. What is the likelihood that the patient will participate in treatment?	☐	☐	☐	☐
c. What is the likelihood that the patient will comply with therapy?	☐	☐	☐	☐
d. What is the likelihood that the patient will sue for malpractice?	☐	☐	☐	☐

For some treatment decisions, patient preferences play little or no role.

5. How likely would you be to ask this patient's preferences for receiving each of the following treatments when you are making a treatment decision?

	Very unlikely	Unlikely	Likely	Very likely
a. Cardiac catheterization	☐	☐	☐	☐
b. Cardiac rehabilitation	☐	☐	☐	☐
c. Beta-blocker	☐	☐	☐	☐
d. Statin	☐	☐	☐	☐
e. ACE inhibitor	☐	☐	☐	☐
f. Coronary-artery bypass surgery	☐	☐	☐	☐

For the following questions, we would like to know more about your assessment of the patient. We would like you to make predictions about the future health risks of the patient.

6. What is your best estimate of this patient's probability of developing **dementia** in the next 5 years? Write your **single** best estimate from 0% to 100%. Please do not write ranges.

_________ % chance of developing dementia in the next 5 years

7. What is your best estimate of this patient's probability of suffering a **stroke** in the next 5 years? Write your **single** best estimate from 0% to 100%. Please do not write ranges.

_________ % chance of suffering a stroke in the next 5 years

8. What is your best estimate of this patient's probability of suffering a **heart attack** in the next 5 years? Write your **single** best estimate from 0% to 100%. Please do not write ranges.

_________ % chance of suffering a heart attack in the next 5 years

Section B: Your Clinical Practice

Please answer the following questions about yourself and your clinical practice.

1. How many MI patients do you estimate you have taken care of in the past 12 months, either as a member of the primary physician team or as a consultant?

   ☐ None
   ☐ 1 to 5
   ☐ 6 to 10
   ☐ 11 to 15
   ☐ 16 to 20
   ☐ 21 or more

2. Please describe your current area(s) of practice specialty.

   ☐ Family Medicine
   ☐ Internal Medicine
   ☐ Cardiology
   ☐ Emergency Medicine
   ☐ Other (specify):____________________

3. Your age _________

4. The year of graduation from medical school _________

5. Gender: ☐ Male ☐ Female

6. Are you board certified? ☐Yes ☐No

7. Are you Hispanic or Latino(a)? ☐Yes ☐No

8. What is your race? (select all that apply)

   ☐ American Indian or Alaska Native
   ☐ East/Southeast Asian or Pacific Islander
   ☐ Black or African American
   ☐ Middle Eastern or Arab American
   ☐ Central/South Asian
   ☐ White, European, or Caucasian
   ☐ Other [Please specify: ____]

9. In which part of the country do you currently practice?

   ☐ Northeast: CT, MA, ME, NH, NJ, NY, PA, RI, VT
   ☐ Midwest: IA, IL, IN, KS, MI, MN, MO, ND, NE, OH SD, WI
   ☐ South: AL, AR, DE, FL, GA, KY, LA, MD, MS, NC, OK, SC, TN, TX, VA, Wash DC, WV
   ☐ West: AK, AZ, CA, CO, HI, ID, MT, NM, NV, OR, UT, WA, WY
   ☐ Other (please specify): ______________________________________

10. Has a close family member or friend had dementia? ☐Yes ☐No

11. In general, how likely would you be to ask patients their preferences for receiving the treatment when you are making the following treatment decisions?

	Very unlikely	Unlikely	Likely	Very likely
a. Cardiac catheterization	☐	☐	☐	☐
b. Cardiac rehabilitation	☐	☐	☐	☐
c. Beta-blocker	☐	☐	☐	☐
d. Statin	☐	☐	☐	☐
e. ACE inhibitor	☐	☐	☐	☐
f. Coronary-artery bypass surgery	☐	☐	☐	☐

Please write any comments about the survey in the space below:

Thank you very much for completing this survey!
