# Supplementary material for: Physician decision-making and recommendations for stroke and myocardial infarction treatments in older adults with mild cognitive impairment
Source: PLoS One. 2020 Mar 17;15(3):e0230446. doi: 10.1371/journal.pone.0230446 (PMC7077853; doi:10.1371/journal.pone.0230446)
Supplement: S2 File — (PDF) [file pone.0230446.s002.pdf]

Physician Perspectives on Stroke Treatment

Sponsored by:

Michigan Alzheimer's Disease Center - Pilot Project Program

Return to:

Dr. Deborah Levine
Departments of Medicine & Neurology
2800 Plymouth Road, Building 16-430W
Ann Arbor, Michigan 48109-2800

Questions? Please call (734) 936-5216 or email deblevin@umich.edu

This study is funded by a grant from the Michigan Alzheimer's Disease Center - Pilot Project Program and has been approved by the Institutional Review Board of the University of Michigan (IRB number HUM00096544).

Physician Perspectives on Ischemic Stroke Treatment

Thank you for taking the time to complete this survey. We are interested in learning **how doctors make recommendations for patients with acute ischemic stroke and perceive patients' health risks**. Your answers are **important** to us.

This survey includes **one example case of acute ischemic stroke**. We are specifically interested in **your opinion** on the case, and what you would recommend to the patient and family. There is **no right or wrong answers**, so please answer as you would if the patient in the case were one of your own. We realize that you may ordinarily use additional information in your clinical decision making that is not presented in the case. Please provide your **one best answer to each question** based on the information provided.

Your answers will be kept **confidential and anonymous.**

When you are completing the survey, feel free to **mark the survey with any comments** about questions or answers that are difficult. There is also **space at the end** to write comments.

Section A: Case

Please read the brief clinical scenario below and answer the questions that follow.

Case

A 75 year-old man presents with the acute onset of left-sided hemiparesis and numbness that started one hour before presentation.

His past medical history is significant for hypertension, current smoking, and osteoarthritis. At a recent clinic visit, he was diagnosed with mild cognitive impairment (Mini-Mental Status Examination score was 26 out of 30). His family reports mild difficulty remembering recent conversations, dates, and appointments. He functions independently and does not require help with dressing, bathing, grocery shopping, or taking medicines, but he uses reminders more.

He takes a daily aspirin. He lives at home with his wife. He is a retired auto worker with Medicare insurance, prescription drug coverage, and Blue Cross–Blue Shield supplemental insurance.

Examination reveals a normal temperature with a blood pressure of 170/100 and a pulse of 90, normal respiratory rate and oxygen saturation of 100%. Eyes are open. He is alert and responsive. He follows commands with the right arm and leg. He has partial paralysis of left lower face. His strength is a 2/5 in left arm and leg with no effort to gravity (limb falls to the bed). His strength is a full 5/5 in the right arm and leg and he has no aphasia. He has left-sided sensory loss and neglect. Blood glucose is normal. Electrocardiogram shows normal sinus rhythm and no ischemia. Platelets and coagulation profile are normal. Renal function is normal. Head CT is normal.

He is diagnosed with an acute ischemic stroke in the right middle cerebral artery territory. The NIHSS score is 12 (face-2; arm weakness-3; leg weakness-3; sensory loss-2; inattention-2). NIHSS (National Institutes of Health Stroke Scale) scores range from 0 to 42 with higher scores indicating greater stroke severity.

---

1. What is the likelihood that the patient will benefit from early intravenous thrombolytic treatment with recombinant tissue plasminogen activator (tPA or alteplase) within 3 hours of stroke onset?

Very unlikely	Unlikely	Likely	Very likely
☐	☐	☐	☐

2. In a patient like this, how likely would you be to…

	Definitely no	Probably no	Probably yes	Definitely yes
Recommend treatment with intravenous tPA within 3 hours of stroke onset	☐	☐	☐	☐

Now imagine that the patient received tPA with some clinical improvement and no complications. The strength in his left arm and leg is improving. You are writing admission orders.

3. In a patient like this, how likely would you be to…

	Definitely no	Probably no	Probably yes	Definitely yes
a. Recommend carotid artery imaging	☐	☐	☐	☐
b. Recommend echocardiogram	☐	☐	☐	☐
c. Admit to stroke unit assuming your hospital had a stroke unit	☐	☐	☐	☐
d. Recommend care by an inpatient stroke team assuming your hospital had a stroke team	☐	☐	☐	☐
e. Recommend brain MRI	☐	☐	☐	☐

Now imagine that the patient had a carotid Doppler showing high-grade right (ipsilateral) carotid artery stenosis of >70% to 99% that was confirmed by CT angiogram. He has no significant left carotid artery stenosis.

4. In a patient like this, how likely would you be to…

	Definitely no	Probably no	Probably yes	Definitely yes
Recommend carotid revascularization (carotid endarterectomy or carotid stenting) at the appropriate timing after stroke and tPA	☐	☐	☐	☐

Now imagine that two days later his strength is a 3/5 in left arm and leg with movement possible against gravity (limb falls to the bed). Left-sided sensory loss and neglect persist. A swallowing study is normal.

His transthoracic echocardiogram shows no thrombus or intra-cardiac shunt, normal left ventricular systolic function, and left atrial dilatation of 44 mm. Telemetry showed normal sinus rhythm. He is medically ready for discharge.

5. In a patient like this, how likely would you be to…

	Definitely no	Probably no	Probably yes	Definitely yes
a. Recommend inpatient rehabilitation	☐	☐	☐	☐
b. Recommend long-term cardiac monitoring (eg, for 21 to 30 days)	☐	☐	☐	☐
c. Recommend statin	☐	☐	☐	☐

6. For the following questions, we would like to know more about your assessment of the patient. Given the limited information that you have we would like you to make predictions about what you think would happen with this patient.

	Very unlikely	Unlikely	Likely	Very likely
a. What is the likelihood that the patient will miss follow-up appointments?	☐	☐	☐	☐
b. What is the likelihood that the patient will participate in treatment?	☐	☐	☐	☐
c. What is the likelihood that the patient will comply with therapy?	☐	☐	☐	☐
d. What is the likelihood that the patient will sue for malpractice?	☐	☐	☐	☐

For some treatment decisions, patient preferences play little or no role.

7. How likely would you be to ask this patient's preferences for receiving each of the following treatments when you are making a treatment decision?

	Very unlikely	Unlikely	Likely	Very likely
a. Thrombolysis with tPA	☐	☐	☐	☐
b. Placement of gastrostomy tube	☐	☐	☐	☐
c. Inpatient rehabilitation	☐	☐	☐	☐
d. Long-term cardiac monitoring (eg, for 21 to 30 days)	☐	☐	☐	☐
e. Statin	☐	☐	☐	☐
f. Oral anticoagulation	☐	☐	☐	☐
g. Carotid revascularization (carotid endarterectomy or carotid stenting)	☐	☐	☐	☐

For the following questions, we would like to know more about your assessment of the patient. We would like you to make predictions about the future health risks of the patient.

8. What is your best estimate of this patient's probability of developing **dementia** in the next 5 years? Write your **<u>single</u>** best estimate from 0% to 100%. Please do not write ranges.

_________ % chance of developing dementia in the next 5 years

9. What is your best estimate of this patient's probability of suffering a **stroke** in the next 5 years? Write your **<u>single</u>** best estimate from 0% to 100%. Please do not write ranges.

_________ % chance of suffering a stroke in the next 5 years

10. What is your best estimate of this patient's probability of suffering a **heart attack** in the next 5 years? Write your **<u>single</u>** best estimate from 0% to 100%. Please do not write ranges.

_________ % chance of suffering a heart attack in the next 5 years

Section B. Your Clinical Practice

Please answer the following questions about yourself and your clinical practice.

1. How many ischemic stroke patients do you estimate you have taken care of in the past 12 months, either as a member of the primary physician team or as a consultant?

   ☐ None
   ☐ 1 to 5
   ☐ 6 to 10
   ☐ 11 to 15
   ☐ 16 to 20
   ☐ 21 or more

2. Please describe your current area(s) of practice specialty.

   ☐ Family Medicine
   ☐ Internal Medicine
   ☐ Cardiology
   ☐ Emergency Medicine
   ☐ Neurology
   ☐ Other (specify):____________________

3. Your age _________

4. The year of graduation from medical school _________

5. Gender: ☐ Male ☐ Female ☐ Other

6. Are you board certified? ☐Yes ☐No

7. Are you Hispanic or Latino(a)? ☐Yes ☐No

8. What is your race? (select all that apply)

   ☐ American Indian or Alaska Native
   ☐ East/Southeast Asian or Pacific Islander
   ☐ Black or African American
   ☐ Middle Eastern or Arab American
   ☐ Central/South Asian
   ☐ White, European, or Caucasian
   ☐ Other [Please specify:

9. In which part of the country do you currently practice?

   ☐ Northeast: CT, MA, ME, NH, NJ, NY, PA, RI, VT
   ☐ Midwest: IA, IL, IN, KS, MI, MN, MO, ND, NE, OH SD, WI
   ☐ South: AL, AR, DE, FL, GA, KY, LA, MD, MS, NC, OK, SC, TN, TX, VA, Wash DC, WV
   ☐ West: AK, AZ, CA, CO, HI, ID, MT, NM, NV, OR, UT, WA, WY
   ☐ Other (please specify): ______________________________________

10. Has a close family member or friend had dementia? ☐Yes ☐No

11. In general, how likely would you be to ask patients their preferences for receiving the treatment when you are making the following treatment decisions?

	Very unlikely	Unlikely	Likely	Very likely
a. Thrombolysis with tPA	☐	☐	☐	☐
b. Placement of gastrostomy tube	☐	☐	☐	☐
c. Inpatient rehabilitation	☐	☐	☐	☐
d. Long-term cardiac monitoring (eg, for 21 to 30 days)	☐	☐	☐	☐
e. Statin	☐	☐	☐	☐
f. Oral anticoagulation	☐	☐	☐	☐
g. Carotid revascularization (carotid endarterectomy or carotid stenting)	☐	☐	☐	☐

Please write any comments about the survey in the space below:

Thank you very much for completing this survey!
