# Supplementary material for: Physician decision-making and recommendations for stroke and myocardial infarction treatments in older adults with mild cognitive impairment
Source: PLoS One. 2020 Mar 17;15(3):e0230446. doi: 10.1371/journal.pone.0230446 (PMC7077853; doi:10.1371/journal.pone.0230446)
Supplement: S3 File — (PDF) [file pone.0230446.s003.pdf]

PHYSICIAN INTERVIEW GUIDE

Study: Pilot Interviews of Physicians for February 2015 R01 Application, HUM00096647
Study PI: Deborah Levine, MD

1. Introduction interviewer and note taker.

Hello, my name is Colleen Dowd Kollman. I am part of the research team. **Thank you** for taking the time to talk with us today.

2. Introduce the project and explain the purpose of the interview.

We are asking for your input because we are **planning new programs** that might improve the way we care for older adults with acute illness, like heart attack or stroke. One of our goals is to better understand how **mild cognitive impairment may affect physician's recommendations for medical treatment.** Mild cognitive impairment is defined as objective cognitive impairment that is not severe enough to cause difficulties with usual activities of daily living such as bathing, dressing, grocery shopping, and taking medicines. **We asked you to talk with us** because you have experience managing patients, some of whom may have mild cognitive impairment.

3. Describe the interview process, assure confidentiality, obtain consent, and answer any questions.

Over the next **60 minutes**, I'll be asking you several questions. There are **no wrong answers**. People have different experiences and points of view, and we want to hear them all.

I want to let you know what will happen to the information you provide. We are **audio-recording** this discussion because we don't want to miss any of your comments. We also may take some **notes** so that we can follow up on something you said later on. Your comments will be kept **confidential**. People working on this study will be the ones who will use the interview recordings. We will take steps to ensure the information you provide remains confidential. These steps are described in the consent form. We will not use individual names in any notes, report, or summary.

Your participation is **voluntary** and you can ask to skip a question or to stop the interview at any time. We will mail you a $100 check or gift card after the interview as a thank you for your participation.

Before we begin, can you please take a few minutes to review and **sign the consent form**? It covers what I have just explained to you. Your signature shows that you understand what the study is about and that you agree to be interviewed and recorded.

Do you have **any questions** for me before you review the consent form (then give **consent form**)?

Physician Interview Guide

The study is designed to learn about how physicians think about treatment recommendations and health risks in adult patients with mild cognitive impairment. Mild cognitive impairment is currently defined as objective cognitive impairment that is not severe enough to cause difficulties with activities of daily living such as bathing, dressing, grocery shopping, and taking medicines.

Big Question #1: How does mild cognitive impairment influence physician recommendations for heart attack and stroke treatment?

Big Question #2: How do physicians perceive the risks of dementia, cancer, cardiovascular disease in adults with mild cognitive impairment?

Introduction

To start, could you tell me about how many patients do you see who have mild cognitive impairment? Now I am also curious about how many patients with mild dementia do you see?

Now think about your patients with mild cognitive impairment. How often do you treat them for illnesses like heart attack or stroke?

General Medical Management

1. Now think only about your patients with mild cognitive impairment. What are some of the challenges you face in caring for these patients? Probes:
    a. Diagnosis
    b. Treatment
    c. Medications
    d. Ascertaining treatment preferences

2. How do these patients compare to your patients with normal cognitive status in terms of your approach to clinical management? How about compared to patient with dementia? Please give me examples if you can. Probes:
    a. What factors you consider in recommending diagnostic tests. Probes:
        i. Goals of care
        ii. Benefits and risks of the test
        iii. How invasive the test is
        iv. Amount of difficulty in educating and communicating the benefits and risks of test
        v. Co-morbidity of the patient
        vi. Level of patient participation in medical decisions and treatment
        vii. Ascertaining patient preferences
        viii. Working with family or friends who may help patient
    b. What factors you consider in deciding about medical treatment. Probes:
        i. Goals of care
        ii. Benefits and risks of the treatment

iii. Intensity of treatment
iv. Amount of difficulty in educating and communicating the benefits and risks of treatment
v. Competing risks of death or other illnesses
vi. Co-morbidity of the patient
vii. Level of patient participation in medical decisions and treatment
viii. Ascertaining patient preferences
ix. Patient's adherence to the treatment
x. Working with family or friends who may help patient
xi. Level of patient participation in treatment

c. How much you involve the patient in deciding about tests and treatments. By involve I mean ask the patient his or her preferences for doing the test or taking the treatment.

3. Can you think of a negative clinical experience you had or know of that involved a patient with mild cognitive impairment? Tell me about that experience. Probes:
    a. The clinical experience could have affected the patient negatively. An example is a medical worker making negative comments about a patient's ability to think.
    b. The clinical experience could have affected the patient's family or friends negatively. An example is a medical worker failing to communicate with the patient's family about an important treatment decision or hospital discharge.
    c. The clinical experience could have affected you or your colleagues negatively. An example is a medical worker questioning the value of doing a test or treatment on a cognitively impaired patient.

4. Can you think of a positive clinical experience you had or know of that involved a patient with mild cognitive impairment? Tell me about that experience. Probes:
    a. The clinical experience could have affected the patient positively. An example is a medical worker taking care to ensure that the patient understands a test result or that the patient's treatment preferences are ascertained.
    b. The clinical experience could have affected the patient's family or friends positively. An example is a medical worker taking care to ensure that the patient's family understands a test result.
    c. The clinical experience could have affected you or your colleagues positively. An example is a patient with MCI having a good clinical outcome or clinical improvement.

Decision-making about Specific Treatments

5. Now thinking about patients with MCI who you have treated, I'd like to ask you about decisions about specific tests and treatments (interviewer asks one by one). Has mild cognitive impairment influenced how you make decisions about the following for patients with MCI? If yes, how?
    a. Surgery
    b. Invasive tests or procedures like catheterization

c. Noninvasive tests like ultrasounds or CT scans
d. Rehabilitation
e. Preventive medicines

6. CARDIOLOGISTS/INTERNISTS: Has the fact that a patient has mild cognitive impairment influenced how you treated him or her for heart attack? If yes, how? Then ask each question below. [KEY QUESTION]
   a. Where you admitted him or her (eg, specialized heart unit or not)
   b. How specialists were involved in the patient's care
   c. Your recommendation for cardiac catheterization
   d. Your recommendation for coronary artery bypass surgery
   e. Your recommendation for cardiac rehabilitation
   f. Your recommendation for a defibrillator
   g. Your recommendation for a statin
   h. Your recommendation for a beta blocker
   i. Your recommendation for an ACE inhibitor
   j. Your recommendation for other heart medicine or treatments

7. NEUROLOGISTS/INTERNISTS: Has the fact that a patient has mild cognitive impairment influenced how you treated him or her for stroke? If yes, how? Then ask each question below. [KEY QUESTION]
   a. Where you admitted him or her (eg, specialized stroke unit or not)
   b. How specialists were involved in the patient's care
   c. Your recommendation for tPA or thrombolysis
   d. Your recommendation for an imaging test of the carotid arteries
   e. Your recommendation for surgery for carotid artery stenosis
   f. Your recommendation for a permanent feeding tube
   g. Your recommendation for a statin
   h. Your recommendation for blood pressure medicine
   i. Your recommendation for oral anticoagulation (eg, in patients with atrial fibrillation)
   j. Your recommendation for outpatient cardiac monitoring for atrial fibrillation

Conceptions of Risk

8. Based on your knowledge and experience, does having MCI affect a patient's risk of dementia?

9. Based on your knowledge and experience, does having MCI affect a patient's risk of having a cardiovascular event like heart attack or stroke?

Support for Managing Patients with MCI

10. What sources of information do you have to help you manage patients with mild cognitive impairment? How helpful are they?
    a. Colleagues

b. Specialists
c. Journal articles
d. Medical society

11. What support would be helpful to you in managing patients with mild cognitive impairment?

Conclusion

12. Is there anything else that you can share with me that might help me better understand the influence of MCI in your clinical experiences? Please feel free to share any stories or specific examples that might bring this to life for me.
